# Alleviation of taurine on lung injury in fatty liver hemorrhagic syndrome laying hens by improving antioxidant and anti-inflammatory capacity

**DOI:** 10.1016/j.psj.2025.106184

**Published:** 2025-12-02

**Authors:** Guangyi Ouyang, Weiwei Li, Wenke Sun, Jishuang San, Meichao Dai, Pingping Wei, Jiancheng Yang, Gaofeng Wu

**Affiliations:** Liaoning Provincial Key Laboratory of Zoonosis, College of Animal Science & Veterinary Medicine, Shenyang Agricultural University, Shenyang, 110866, China

**Keywords:** Taurine, Oxidative stress, Inflammatory reaction, Lung injury, Fatty liver hemorrhagic syndrome laying hens

## Abstract

Fatty liver hemorrhagic syndrome (FLHS), a common metabolism-associated fatty liver disease (MAFLD) in modern intensive laying hens, affects not only liver function but also other organs, including the lungs, causing lung impairment and susceptibility to respiratory ailments. Clinical investigations have revealed an independent association between MAFLD and diminished lung function. Previous studies have demonstrated that taurine enhances the body's antioxidant capacity and exerts a significant inhibitory effect on both acute and chronic inflammation. Additionally, taurine exhibits promising preventive and therapeutic efficacy in the occurrence and development of FLHS, significantly ameliorating liver damage in laying hens. However, whether taurine can mitigate FLHS-induced lung injury remains unexplored. This study investigated the effects of taurine on lung injury in FLHS laying hens induced by a high-energy and low-protein diet, and macrophage inflammation model stimulated by Lipopolysaccharide (LPS). The results showed that taurine treatment significantly reduced inflammatory cell infiltration in lung tissue and improved alveolar structure. Furthermore, antioxidant enzyme activities such as superoxide dismutase (SOD), catalase (CAT), glutathione peroxidase (GSH-Px), as well as the nuclear factor erythroid 2-related factor 2 (Nrf2) signaling pathway and its downstream factors expression levels of quinone oxidoreductase 1 (NQO1) and heme oxygenase-1 (HO-1), were significantly increased in the lungs and macrophages of laying hens following taurine administration. Taurine also effectively suppressed M1 macrophage polarization, reduced levels of proinflammatory cytokines, including inducible nitric oxide synthase (iNOS), nitric oxide (NO), interleukin-1β (IL-1β), and tumor necrosis factor-α (TNF-α), inhibited the activation of the LPS/TLR4/NF-κB signaling pathway, JAK2-STAT1 signaling pathway, and NLRP3 inflammasome. The results suggested that taurine can alleviate lung injury in FLHS laying hens by enhancing antioxidant capacity and inhibiting inflammatory response mediated by macrophage M1 polarization.

## Introduction

Fatty liver hemorrhagic syndrome (FLHS) is a metabolic disorder characterized by dysregulated lipid metabolism, with a worldwide incidence of up to 16 % and potentially reaching 30 % in severe cases. FLHS commonly affects laying hens during peak egg production, leading to liver enlargement, hemorrhaging, and necrosis. These conditions often result in decreased egg production, increased mortality rates(Chen et al., 2021; Meng et al., 2021; You et al., 2023), and substantial economic losses in the poultry industry, significantly impacting its growth. The etiology of FLHS may stem from imbalances in lipid metabolism influenced by factors such as nutrition, environment, hormones, and genetics, although its precise pathogenesis remains unclear. FLHS shares similarities with metabolic-associated fatty liver disease (MAFLD) in terms of etiology and clinical manifestations, suggesting a potential common pathogenic mechanism known as the "Two-hit Hypothesis"(Jian et al., 2022; Li et al., 2023). The first hit involves excessive accumulation of fat in the liver due to impaired lipolysis and fat production, while the second hit is associated with redox imbalance and inflammation. Excessive accumulation of lipids in the liver stimulated the production of reactive oxygen species (ROS), which induced oxidative stress and inflammation in cells. During MAFLD progression, intestinal microbial metabolism disorder leads to intestinal barrier destruction, allowing intestinal microbial products such as lipopolysaccharide (LPS) to migrate into the blood, which can cause oxidative stress and inflammation in the liver. MAFLD not only affects liver function, but also causes lung damage (Byrne and Targher, 2015; Targher et al., 2024), which predisposes laying hens to respiratory tract infections and exacerbates FLHS-related conditions.

Taurine, a sulfur-containing free amino acid, derives its name from its initial discovery and isolation from bovine bile. It is abundantly present in the body and exerts various physiological and pharmacological effects, encompassing antioxidant, anti-inflammatory, osmotic regulation, calcium homeostasis, bile salt formation, and central nervous system functions (Lambert et al., 2015; Lin et al., 2025). As an oxygen free radical scavenger, taurine modulates the antioxidant system, effectively mitigating oxidative stress (Maleki et al., 2020; Wang et al., 2024). Numerous studies have demonstrated that taurine enhances the activity of antioxidant enzymes such as superoxide dismutase (SOD), catalase (CAT), and glutathione peroxidase (GSH-Px), elevates total antioxidant capacity (T-AOC), maintains redox balance, and alleviates oxidative stress by activating the nuclear factor erythroid 2-related factor 2 (Nrf2) signaling pathway and downstream antioxidant enzymes (Baliou et al., 2021; Faghfouri et al., 2022; Murakami et al., 2022; Shi et al., 2022). Moreover, taurine can diminish levels of inflammatory factors like interleukin-1β (IL-1β) and tumor necrosis factor-α (TNF-α) in lung tissue by suppressing the p38/MAPK and NF-κB signaling pathways, thereby attenuating sepsis-induced lung injury (Chen et al., 2021). In a rat acute lung injury model, taurine could reduce the content of pro-inflammatory cytokines IL-1β and interleukin-6 (IL-6), alleviate pulmonary inflammatory response, and demonstrate therapeutic potential for acute lung injury(Zhao et al., 2018). Therefore, we hypothesized that taurine might alleviate lung injury in FLHS laying hens by alleviating oxidative stress and inflammatory responses. In this experiment, FLHS in laying hens was induced by feeding high-energy and low-protein diets, and macrophages were cultured in vitro to establish an inflammation model. Then the taurine was administered concurrently to evaluate its effect. We examined how taurine influences lung antioxidant capacity and macrophage function. Specifically, we assessed its impact on macrophage M1-type polarization and the associated inflammatory response in FLHS. Our findings reveal the protective role of taurine against FLHS-induced lung injury. This work elucidates a potential mechanism underlying taurine’s protective effects and provides a theoretical foundation for its use in preventing lung damage in FLHS.

## Materials and methods

### Animals and experimental design

In this experiment, 48 23-week-old healthy Hyland Brown laying hens (mean weight 1.5 kg ± 0.2 kg) with similar body weight and egg production rate and at peak laying stage were selected and caged in 3 layers. During the experimental period, the hens were fed twice a day with ad libitum water. Feeding conditions were temperature (18 ∼ 21°C) and humidity (40 % ∼ 70 %) with a light-dark cycle of 16-8 h. After a one-week acclimatization period, the laying hens were randomly divided into three groups: the normal control group (Con) fed a standard maize-soya bean meal-based basal diet, the FLHS model (FLHS) group fed a high-energy and low-protein diet, and the taurine intervention group was fed a high-energy and low-protein diet with 0.05 % taurine. The feed composition ratio was referred to the results of the previous study of the group (San et al., 2023). After 20 weeks of feeding, all laying hens were executed, the blood, liver and lungs were collected for further analysis. This study was reviewed and approved by the Animal Care and Use Committee of Shenyang Agricultural University (2021060103).

### Cell culture and treatment

RAW264.7 cells were purchased from the cell bank of Shanghai Institute of Cell Biology and Biochemistry, Chinese Academy of Sciences (Shanghai, China). The cells were maintained in DMEM containing 100 U/ml penicillin, 100 μg/ml streptomycin, 2 mM glutamine and 10 % fetal bovine serum, and cultured in a humid environment at 37 °C with 5 % CO_2_. Passage culture or subsequent experiments were performed when the cell fusion reached 70-80 %. Referring to previous studies, the macrophage inflammation model was established by treating with 1 μg/mL LPS in serum-free medium for 24 h (Peng et al., 2024). Meanwhile, taurine was added to the serum-free medium and cultured at 37 °C for 24 h. The cells were divided into three groups, namely, the normal control group (Con), the inflammation model group (LPS), and the taurine intervention group (LPS+T).

### Cell viability assay

RAW264.7 cells were inoculated in 96-well plates and different treatments were performed when the cells grew to 80 % fusion. Then, cell viability was determined by Cell Counting Kit-8 (CCK-8; using Solarbio, Beijing, China). First, 10 µL of CCK-8 detection reagent was added to each well and incubated at 37°C for 4 hours. Finally, the absorbance of the solution was assessed at 450 nm using an enzyme marker (Infinite M200PRO, TECAN, Shanghai, China).

### Biochemical index analyses

All biochemical analyses were performed using commercial kits (Nanjing Jiancheng Bioengineering Institute, Nanjing, China). Liver function indicators, including alanine aminotransferase (ALT) (C009-2-1) and aspartate aminotransferase (AST) (C010-2-1); lung and RAW264.7 Cellular antioxidant capacity indicators, including total superoxide dismutase (T-SOD) (A001-3-2), catalase (CAT) (A007-1-1), glutathione peroxidase oxidase (GSH-Px) (A005-1-2) and malondialdehyde (MDA) (A003-4-1). The kit instructions were followed and absorbance was measured using an enzyme marker (Infinite M200PRO, TECAN, Shanghai, China).

### Inflammatory factor assays

All assays were performed using commercially available kits (Nanjing Jiancheng Bioengineering Institute, Nanjing, China). The inflammatory factors included: interleukin-1β (ΙL-1β) (Η002-1-2), inducible nitric oxide synthase (iNOS) (H371-1-2), and nitric oxide (NO) (A013-2-1). The kit instructions were followed and absorbance was measured using an enzyme marker(Infinite M200PRO, TECAN, Shanghai, China).

### Hematoxylin and eosin (H&E) staining

Laying hen liver and lung tissue blocks fixed in 4 % paraformaldehyde specimen vials were removed. Liver and lung tissue sections were made according to the steps of rinsing, dehydrating and clearing, dipping and embedding in wax, and slicing and gluing, according to the instructions of the HE staining kit. After staining, the sections were sealed using a neutral tree glue, and after waiting for the sections to completely dry out, the sections were examined and photographed under 200× magnification using a Leica inverted microscope (DM4000B) at 200× magnification to examine and photograph the sections.

### Real-time quantitative PCR (RT-qPCR)

Total RNA was obtained from lungs and RAW264.7 cells using a total RNA extraction kit (TIANGEN, Beijing, China, DP430). cDNA was then synthesised using a ChamQ Universal SYBR qPCR Master Mix (Vazyme Biotechnology Co., Ltd. Nanjing, China, R323-01) according to the manufacturer's instructions. HiScript III RT SuperMix and gDNA wiper to synthesise cDNA. cDNA was quantified by RT-qPCR using ChamQ Universal SYBR qPCR Master Mix (Vazyme Biotechnology Co., Ltd. Nanjing, China, Q711-02) to detect the relevant. The primer sequences used for RT-qPCR are shown in Table 1. 2-∆∆Ct method was used to calculate the relative gene expression levels. The results are shown as the relative fold change of the control normalised to the endogenous control β-actin or GAPDH values.

**Table 1:** RT-qPCR primer sequences.

Gene	Primer sequences	GenBank Accession number	Product size(bp)
Gallus- Nrf2	F: GATGTCACCCTGCCCTTAG R: CTGCCACCATGTTATTCC	NM_205117.1	215
Gallus- HO-1	F: GTCGTTGGCAAGAAGCATCC R: GGGCCTTTTGGGCGATTTTC	NM_205344.2	106
Gallus- NQO1	F: CGAGTGCTTTGTCTACGAGATGGAG R: AGGTCAGCCGCTTCAATCTTCTTC	NM_001277620.2	102
Gallus- CD80	F: TCGTTCAGAGTCTCCAGTCTTCACC R: CAGCGGTAACAAAGAGGGTCACAG	XM_048958972.1	84
Gallus- iNOS	F: GGAACAGAGATTGGAGTGCGAGAC R: CTGGTGGAACACGGGAGTTATGC	NM_204961.2	310
Gallus- JAK2	F: GTGTGGAGATGTGCCGCTATG R: AGTGCTGTGCTGGAGTTTCTTC	NM_001048177.3	80
Gallus- STAT1	F: GGCGAAGAGCGACCAGAAAC R: AGCTGATCCAGGCAGGCATT	NM_032612.3	216
Gallus- TLR4	F: GGAGGTTGTAGATTTGAGTGAG R: AGATGGGACATAACATGAGTTT	NM_001030693.2	270
Gallus- MyD88	F: CCTGGCTGTGCCTTCGGA R: TCACCAAGTGCTGGATGCTA	XM_046910878.1	198
Gallus- NF-κB	F: CAGCCCATCTATGACAACCG R: CAGCCCAGAAACGAACCTC	NM_001396038.1	151
Gallus- LBP	F: GAGCCGGAAGGTTATTGTGGTAGTG R: AGGACAATGAAGATGATGCCAGAGC	XM_004947186.5	126
Gallus- IRF5	F: TTCAAGGCATGGGCAACAGAGAC R: CGTCCTCTTCCTCCTCCTCTTCC	XM_046908601.1	250
Gallus- NLRP3	F: GCTCCTTGCGTGCTCTAAGACC R: TTGTGCTTCCAGATGCCGTCAG	NM_001348947.1	150
Gallus- Caspase-1	F: GTGCTGCCGTGGAGACAACATAG R: AGGAGACAGTATCAGGCGTGGAAG	XM_025142104.1	179
Gallus- Caspase-11	F: GGCTACGATGTGGTGGTGAAAGAG R: ATGTGCTGTCTGATGTCTGGTGTTC	NM_001379318.1	103
Gallus- GAPDH	F: GAGAGGGAAATCGTGCGTGACA R: CGATAGTGACCTGACCGTCA	NM_031144.3	136
Mouse- Nrf2	F: GTAGATGACCATGAGTCGCTTGCC R: CTTGCTCCATGTCCTGCTCTATGC	XM_032903520.1	148
Mouse- HO-1	F: TGCCAGTGCCACCAAGTTCAAG R: TGTTGAGCAGGAACGCAGTCTTG	XM_033232766.1	116
Mouse- NQO1	F: GGAAGCTGCAGACCTGGTGA R: CCTTTCAGAATGGCTGGCA	XM_036153810.1	69
Mouse- CD86	F: ATCTGCCGTGCCCATTTACA R: CAACTTTTGCTGGTCCTGCC	NM_019388.3	80
Mouse- IL-1β	F: CTCGCAGCAGCACATCAACAAG R: CCACGGGAAAGACACAGGTAGC	XM_006498795.5	94
Mouse- TNF-α	F: CATCTTCTCAAAATTCGAGTGACAA R: TGGGAGTAGACAAGGTACAACCC	NM_013693.3	175
Mouse- iNOS	F: ACTCAGCCAAGCCCTCACCTAC R: TCCAATCTCTGCCTATCCGTCTCG	NM_001313921.1	111
Mouse- LBP	F: GAGCCGGAAGGTTATTGTGGTAGTG R: AGGACAATGAAGATGATGCCAGAGC	NM_008489.2	126
Mouse- CD14	F: GTGTCGTGGGCAACAAGGGATG R: CTAAGGTGGAGAGGGCAGGGAAG	NM_009841.4	145
Mouse- TLR4	F: GGACTCTGATCATGGCACTG R: CTGATCCATGCATTGGTAGGT	NM_021297.3	101
Mouse- MyD88	F: TATACCAACCCTTGCACCAAGTC R: TCAGGCTCCAAGTCAGCTCATC	NM_010851.3	121
Mouse- IRAK1	F: CCTCTGCCTCCACCTTCCTCTC R: GCTCTCTGGGCTTGACTGCTTG	NM_001177973.1	149
Mouse- TRAF6	F: GACTGCCCAACAGCTCCAATCC R: AAGTGTCGTGCCAAGTGATTCCTC	NM_001303273.1	86
Mouse- TAK1	F: TCATCAACAAGCACCACCGTAACC R: ACATCAAAGGGCTTCCGTTCACTC	NM_001410482.1	109
Mouse- NF-κB	F: AAATGGGAAACCGTATGAGCCTGTG R: GTTGTAGCCTCGTGTCTTCTGTCAG	NM_001410442.1	92
Mouse- COX-2	F: AGAAGGAAATGGCTGCAGAA R: GCTCGGCTTCCAGTATTGAG	NM_011198.5	194
Mouse- NLRP3	F: GCTGCGATCAACAGGCGAGAC R: CCATCCACTCTTCTTCAAGGCTGTC	XM_036156549.1	124
Mouse- Caspase-1	F: ATACAACCACTCGTACACGTCTTGC R: TCCTCCAGCAGCAACTTCATTTCTC	NM_009807.2	115
Mouse- Caspase-11	F: GGCTACGATGTGGTGGTGAAAGAG R: ATGTGCTGTCTGATGTCTGGTGTTC	NM_001379322.1	103
Mouse- β-actin	F: GAGAGGGAAATCGTGCGTGACA R: CGATAGTGACCTGACCGTCA	NM_031144.3	136

### Western blot

RAW264.7 cells were lysed using RIPA lysate (Beyotime, Shanghai, China) supplemented with 1 % protease inhibitor mixture (Beyotime, Shanghai, China, P1005). Protein concentration was determined by BCA method. Equal amounts (20 µg) of total proteins were separated by 10 % sodium dodecyl sulfate-polyacrylamide gel electrophoresis (SDS-PAGE) and then electro transferred onto polyvinylidene difluoride (PVDF) membranes. The membranes were closed at 37 degrees for 20-30 min in a protein-free rapid closure solution, and then the membranes were incubated with β-actin (dilution 1:10,000, Immunoway, Plano, TX, USA, YT0099), Toll-like receptor 4 (TLR4) (dilution 1:1000, Immunoway, Plano, TX, USA, YT0744), p-P65 (dilution 1:1000, Immunoway, Plano, TX, USA, YP0191), P65 (dilution 1:2000, Immunoway, Plano, TX, USA, YM3111), NOD-like receptor protein 3 (NLRP3) (dilution 1:1000, Immunoway, Plano, TX, USA, YT5382), Caspase-1 (dilution 1:500, Immunoway, Plano, TX, USA, YT5743) at 4°C overnight. The PVDF membrane was washed three times with TBST for 10 min each time and then incubated with horseradish peroxidase-coupled secondary antibody (dilution 1:20,000, Immunoway, Plano, TX, USA, RS0002) for 1h at 37 degrees. The bands were visualised using an enhanced chemiluminescence direct labelling (ECL) system with β-actin levels as a control. Stained proteins were quantified by optical density using ImageJ (v1.5.3s) software (National Institutes of Health, Bethesda, MD, USA).

### Statistical analyses

All statistical analyses were performed using IBM SPSS23.0 statistical software. One-way analysis of variance (ANOVA) and LSD post hoc tests were used to compare differences between groups. Data were expressed as mean ± standard error of the mean (SEM). Differences were considered significant at *p* < 0.05.

## Results

### Liver function and assessment of liver injury and lung injury

**Fig. 1:**
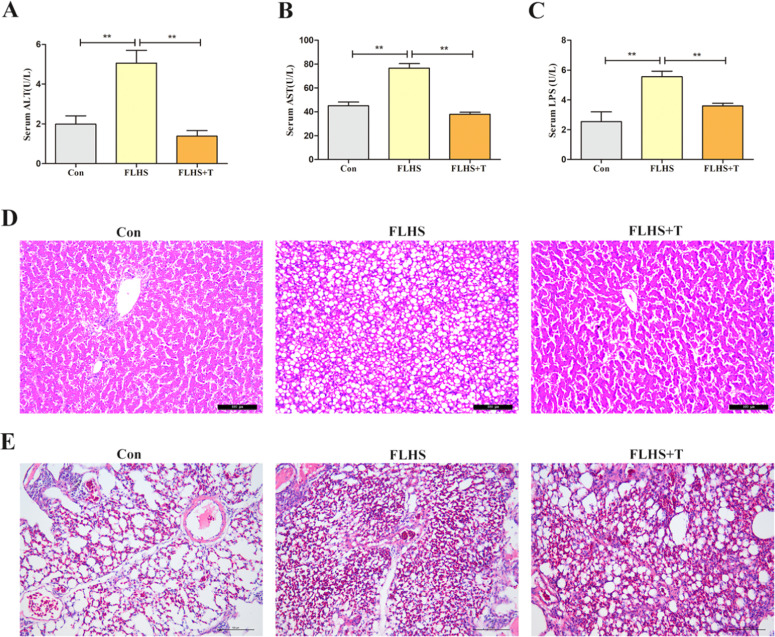
Effects of taurine on liver function and liver injury and lung injury in FLHS laying hens. (A) Results of serum ALT level in FLHS laying hens. (B) Results of serum AST level in FLHS laying hens. (C) Results of serum LPS level in FLHS laying hens. (D) Results of liver tissue section (200X) in FLHS laying hens. (E) Results of lung tissue section (200X) in FLHS laying hens. Data are expressed as mean ± SEM. *P < 0.05 and **P < 0.01.

ALT and AST are important indicators for evaluating liver function, and their release into the bloodstream when the liver is damaged results in elevated serum levels of ALT and AST. The serum levels of ALT and AST in the test laying hens are shown in Fig. 1A and 1B, which were significantly elevated in the FLHS model group compared with the normal control group (*p* < 0.01), while their levels were significantly reduced by the addition of taurine (*p* < 0.01).

The results of HE staining of paraffin sections of laying hens' livers were shown in Fig. 1. D. The morphology of hepatocytes in the normal control group was normal and the hepatocyte cords were clear; in the FLHS model group, the morphology of hepatocytes was disorganized compared with that of the normal control group and some of the hepatocytes showed the phenomenon of nucleolysis, and a large number of fat vacuoles were appeared; the morphology of hepatocytes in the taurine intervention group was significantly restored compared with that of the FLHS model group and the number of fat vacuoles was reduced significantly.

The results of HE staining of paraffin sections of the lungs of laying hens were shown in Fig. 1F. The alveolar morphology was normal in the normal control group, and there was no inflammatory cell infiltration; in the FLHS model group, the lung tissue cavity was enlarged, the alveolar structure was damaged, the structure was incomplete, and there was a large number of inflammatory cells infiltration; in the taurine intervention group, the inflammatory cell infiltration was alleviated compared with that of the FLHS model group, the alveolar structure was intact, and it was basically restored to normal.

As shown in Fig. 1C, the serum LPS levels were significantly higher in the FLHS model group compared with the normal control group (*p* < 0.01), and significantly lower after the addition of taurine (*p* < 0.01).

### Effect of taurine on antioxidant capacity of lungs of FLHS laying hens

**Fig. 2:**
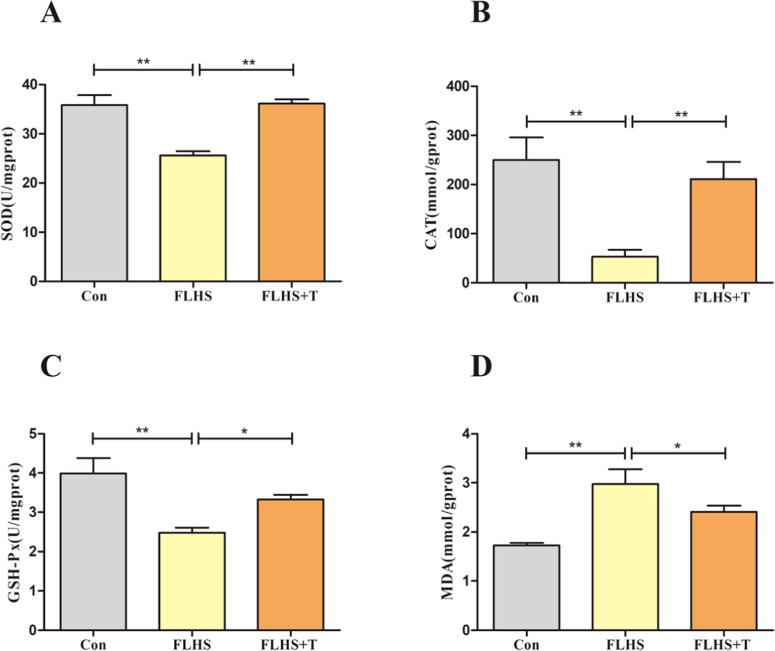
Effect of taurine on antioxidant capacity of lungs of FLHS laying hens. (A) Results of SOD levels in lungs of FLHS laying hens. (B) Results of CAT levels in lungs of FLHS laying hens. (C) Results of GSH-Px levels in lungs of FLHS laying hens. (D) Results of MDA levels in lungs of FLHS laying hens. Data are expressed as mean ± SEM. *P < 0.05 and **P < 0.01.

The results of lung antioxidant indexes in laying hens are shown in Fig. 2A-C, the SOD, CAT and GSH-Px contents were significantly lower (*p* < 0.01) in the FLHS model group compared with the normal control group, and their contents were significantly higher (*p* < 0.05) after the intervention of adding taurine. The lung MDA content of laying hens is shown in Fig. 2D, and its content was significantly higher (*p* < 0.01) in the FLHS model group compared than the normal control group, and its content was significantly lower (*p* < 0.05) after the intervention of adding taurine.

**Fig. 3:**
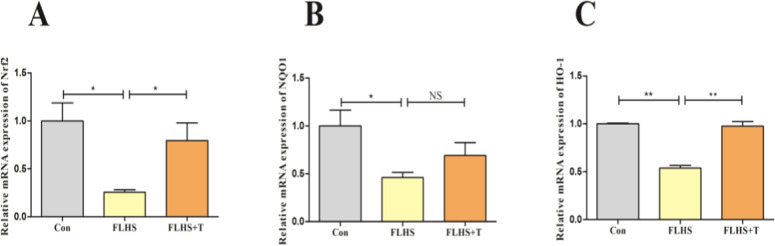
Effect of taurine on Nrf2 signaling pathway in lungs of FLHS laying hens. (A) Detection results of lung Nrf2 mRNA levels in FLHS laying hens. (B) Detection results of lung NQO1 mRNA levels in FLHS laying hens. (C) Detection results of lung HO-1 mRNA levels in FLHS laying hens. Data are expressed as mean ± SEM. *P < 0.05, **P < 0.01, NS (P > 0.05).

In order to further investigate the pathway of taurine to improve the antioxidant capacity of lungs of FLHS laying hens, we examined the indicators related to the Nrf2 antioxidant pathway (Fig. 3). The Nrf2, NQO1, and HO-1 mRNA expression levels were significantly reduced in the FLHS model group compared with the normal control group (*p* < 0.05), and after the intervention of adding taurine, the Nrf2, HO-1 mRNA expression levels increased significantly (*p* < 0.05), and NQO1 mRNA expression levels showed an increasing trend but not significant (p>0.05).

### Effect of taurine on lung inflammatory response in FLHS laying hens

**Fig. 4:**
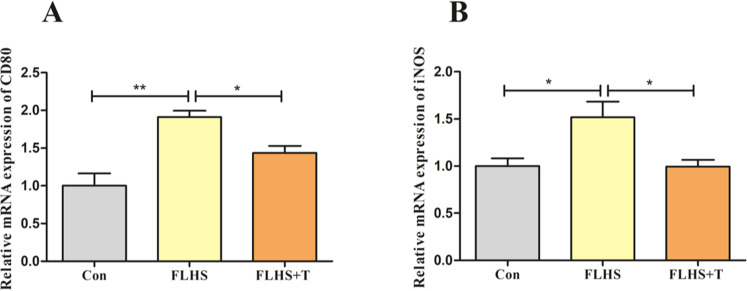
Effect of taurine on M1-type macrophage markers in lungs of FLHS laying hens. (A) Results of CD80 mRNA levels in lungs of FLHS laying hens. (B) Results of iNOS mRNA levels in lungs of FLHS laying hens. Data are expressed as mean ± SEM. *P < 0.05 and **P < 0.01.

Causing lung injury in FLHS laying hens may be related to macrophage infiltration in the lungs in addition to oxidative stress in the lungs. When lung macrophages are exposed to external stimuli such as lipopolysaccharide (LPS), they polarize to M1-type macrophages and release large amounts of pro-inflammatory cytokines. Therefore, the M1-type macrophage markers were examined. As shown in Fig. 4, the expression levels of CD80 and iNOS mRNA were significantly increased in the FLHS model group compared with the normal control group (*p* < 0.05), and the intervention with the addition of taurine resulted in a significant decrease in the expression levels of CD80 and iNOS mRNA (*p* < 0.05).

**Fig. 5:**
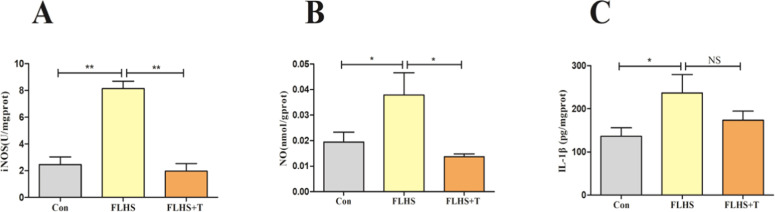
Effect of taurine on lung inflammatory factors in FLHS laying hens. (A) Results of lung iNOS content in FLHS laying hens. (B) Results of lung NO content in FLHS laying hens. (C) Results of lung IL-1β content in FLHS laying hens. Data are expressed as mean ± SEM. *P < 0.05, **P < 0.01, NS (P > 0.05).

Subsequently, the content of pro-inflammatory factors iNOS, NO, and IL-1β produced by M1-type macrophages after activation were examined. The results are shown in Fig. 5, iNOS, NO, and IL-1β contents were significantly increased in the FLHS model group than the normal control group (*p* < 0.05). After the intervention of adding taurine, the content of iNOS, and NO were significantly decreased (*p* < 0.05), but the IL-1 β content showed a decreasing trend but no statistical difference (p > 0.05).

### Effect of taurine on LPS/TLR4/NF-κB signaling pathway in lungs of FLHS laying hens

**Fig. 6:**
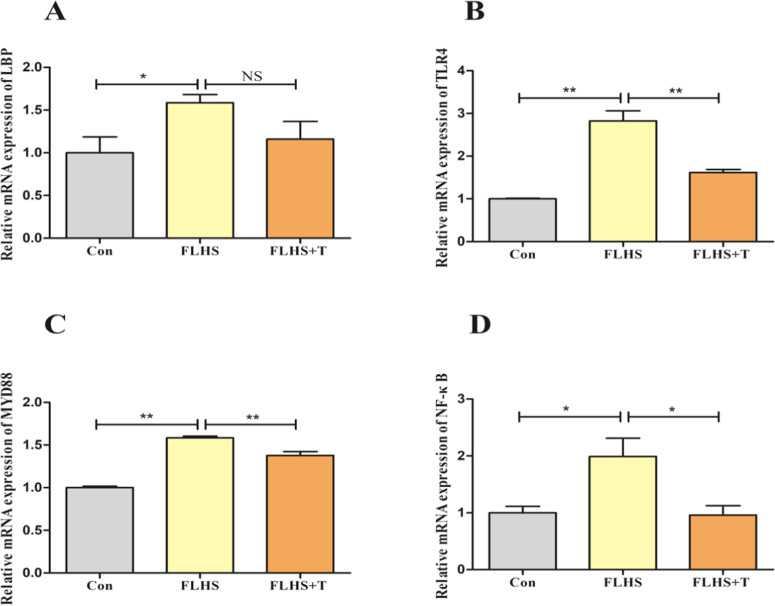
Effect of taurine on LPS/TLR4/NF-κB signaling pathway in lungs of FLHS laying hens. (A) Results of the detection of LBP mRNA expression level in the lungs of FLHS laying hens. (B) Results of the detection of TLR4 mRNA expression level in the lungs of FLHS laying hens. (C) Results of the detection of MYD88 mRNA expression level in the lungs of FLHS laying hens. (D) Results of the detection of NF-κB mRNA expression level in the lungs of FLHS laying hens. Data are expressed as mean ± SEM. *P < 0.05, **P < 0.01, NS (P > 0.05).

LPS/TLR4/Nuclear factor kappa-B (NF-κB) is a signaling pathway that regulates the inflammatory response and plays an important role in the inflammatory response. The results are shown in Fig. 6. And the mRNA expression levels of Lipopolysaccharide Binding Protein (LBP), TLR4, MyD88, and NF-κB were significantly higher in the FLHS model group compared with the normal control group (*p* < 0.05), and after the intervention of adding taurine, the mRNA expression level of LBP showed a decreasing trend but no statistical difference was observed (p > 0.05). The mRNA expression levels of TLR4, MyD88, and NF-κB were significantly reduced (*p* < 0.05).

### Effect of taurine on JAK2-STAT1 signaling pathway in lungs of FLHS laying hens

**Fig. 7:**
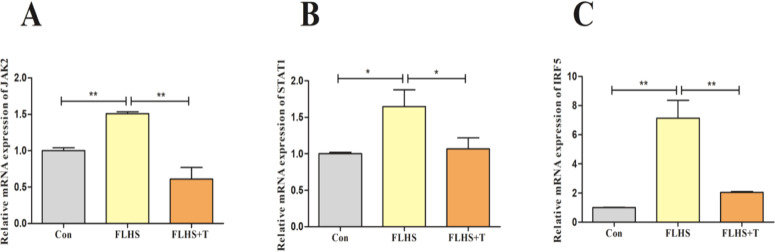
Effects of taurine on JAK2-STAT1 signaling pathway in lungs of FLHS laying hens. (A) Results of JAK2 mRNA expression level detection in the lungs of FLHS laying hens. (B) Results of STAT1 mRNA expression level detection in the lungs of FLHS laying hens. (C) Results of IRF5 mRNA expression level detection in the lungs of FLHS laying hens. Data are expressed as mean ± SEM. *P < 0.05 and **P < 0.01.

The JAK2-STAT1 signaling pathway is an important pathway in the regulation of macrophage polarization to M1 type. Therefore, the factors related to the Janus Kinase 2 (JAK2)-Signal transducer and activator of transcription 1 (STAT1) signaling pathway were examined, and the results are shown in Fig. 7. The mRNA expression levels of JAK2, STAT1, and Interferon Regulatory Factor 5 (IRF5) were significantly higher in the FLHS model group compared with the normal control group (*p* < 0.05). The addition of taurine intervened to significantly reduce the mRNA expression levels of JAK2, STAT1, and IRF5 (*p* < 0.05).

### Effect of taurine on lung NLRP3 inflammatory in FLHS laying hens

**Fig. 8:**
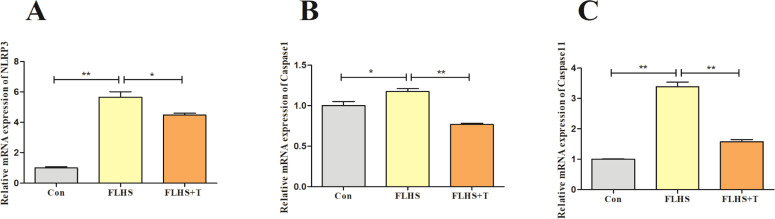
Effect of taurine on NLRP3 inflammatory in lungs of FLHS laying hens. (A) Results of NLRP3 mRNA expression level assay in the lungs of FLHS laying hens. (B) Results of Caspase-1 mRNA expression level assay in the lungs of FLHS laying hens. (C) Results of Caspase-11 mRNA expression level assay in the lungs of FLHS laying hens. Data are expressed as mean ± SEM. *P < 0.05 and **P < 0.01.

Previous studies have shown that NLRP3 inflammatory are significantly activated and mediate inflammatory injury in lung-injured tissues and play an important role in the development of lung injury. Therefore, the results are shown in Fig. 8. The mRNA expression levels of NLRP3, Caspase-1, and Caspase-11 were highly significantly elevated in the FLHS model group compared with the normal control group (P < 0.01); after the addition of taurine intervention, the mRNA expression levels of NLRP3, Caspase-1, and Caspase-11 were significantly elevated, Caspase-11 mRNA expression levels were significantly reduced (P < 0.05).

### In vitro screening of taurine and LPS concentrations

**Fig. 9:**
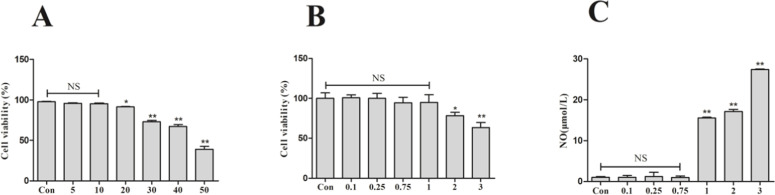
Concentrations of taurine and LPS on RAW264.7 cell line. (A) Results of different concentrations of taurine on the viability of RAW264.7 cells. (B) Results of different concentrations of LPS on the viability of RAW264.7 cells. (C) Results of different concentrations of LPS on the NO release of RAW264.7 cells. Data are expressed as mean ± SEM. *P < 0.05, **P < 0.01, NS (P > 0.05).

As shown in Fig. 9A, we applied different concentration gradients (0,5, 10, 20, 30, 40, 50 mmol/L) of taurine to RAW264.7 cells for 24 h, and detected the cell survival rate of each group by CCK-8 assay, and found that there was a slight increase in the cell survival rate when taurine was applied to 5 and 10 mmol/L, but there was no significant difference compared with that of the normal control group (0 mmol/L). Starting from the 20 mmol/L concentration, the cell survival rate began to decrease significantly (P < 0.01) compared with that of the normal control group. Therefore 10 mmol/L was chosen as the action concentration of taurine for subsequent experiments.

The LPS with 1μg/mL was added to RAW264.7 cells for 24 h. Detection of cell survival by CCK-8 assay revealed that there was no significant difference in cell survival by this concentration (P > 0.05). As shown in Fig. 9, Fig. 9 the NO release from RAW264.7 cells after LPS action was examined, and the cellular NO release was significantly elevated when the LPS action concentration was greater than 1μg/mL (P < 0.01), so we chose 1 μg/mL as the action concentration of LPS.

### Effect of taurine on the antioxidant capacity of RAW264.7 cells

**Fig. 10:**
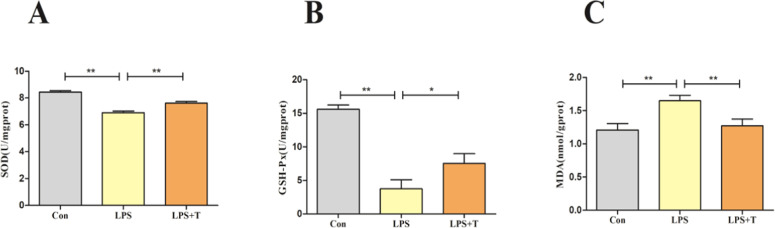
Effect of taurine on antioxidant indexes of RAW264.7 cells. (A) Results of SOD content of RAW264.7 cells. (B) Results of GSH-Px content of RAW264.7 cells. (C) Results of MDA content of RAW264.7 cells. Data were expressed as mean ± SEM. *P < 0.05 and **P < 0.01.

After treating RAW264.7 cells with 1 μg/mL LPS and 10 mmol/L taurine, the cellular antioxidant-related indexes were examined, as shown in Fig. 10, the SOD and GSH-Px contents of the LPS group were significantly decreased (*p* < 0.01) and the MDA content was significantly increased (*p* < 0.01) compared with the normal control group; the addition of taurine intervention significantly increased (*p* < 0.05) the SOD and GSH-Px content significantly increased (*p* < 0.05) and MDA content significantly decreased (*p* < 0.01) after the addition of taurine intervention.

**Fig. 11:**
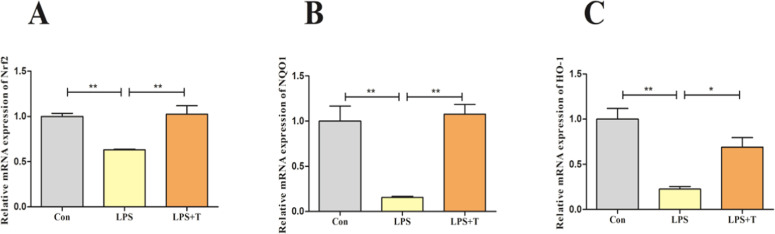
Effects of taurine on antioxidant pathways in RAW264.7 cells. (A) Detection results of Nrf2 mRNA expression level in RAW264.7 cells. (B) Detection results of NQO1 mRNA expression level in RAW264.7 cells. (C) Detection results of HO-1 mRNA expression level in RAW264.7 cells. Data were expressed as mean ± SEM. *P < 0.05 and **P < 0.01.

Then, the Nrf2 antioxidant pathway-related indexes were examined, and the results were consistent with the in vivo test, as shown in Fig. 11, the Nrf2, HO-1, and NQO1 mRNA expression levels were significantly reduced in the LPS group compared with the normal control group (*p* < 0.01), and the Nrf2, HO-1, and NQO1 mRNA expression levels were significantly elevated after the addition of taurine intervention (*p* < 0.05).

### Effect of taurine on the inflammatory response of RAW264.7 cells

**Fig. 12:**
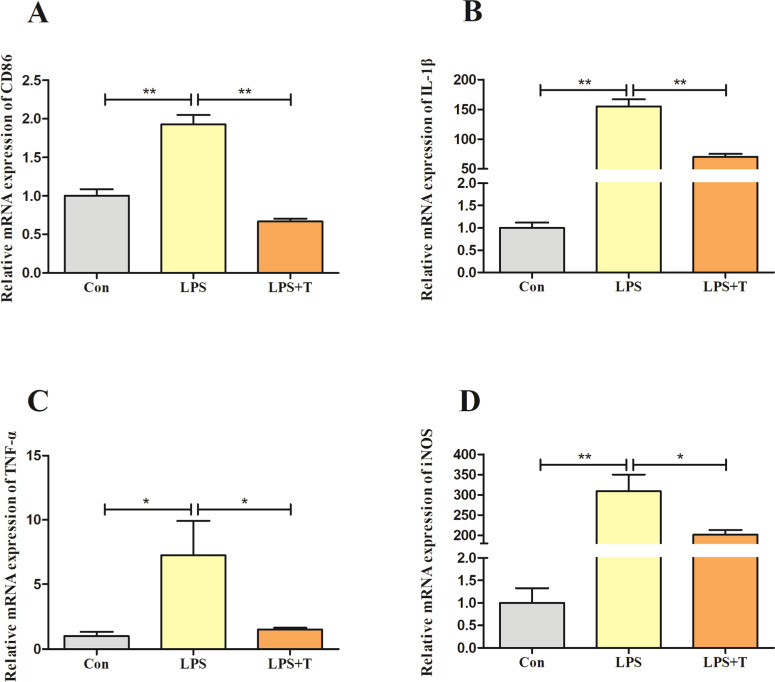
Effect of taurine on the inflammatory response of RAW264.7 cells. (A) Detection results of CD86 mRNA expression level of M1-type polarization marker in RAW264.7 cells. (B) Detection results of IL-1β mRNA expression level in RAW264.7 cells. (C) Detection results of TNF-α mRNA expression level in RAW264.7 cells. (D) Detection results of iNOS mRNA expression level in RAW264.7 cells. Detection results. Data were expressed as mean ± SEM. *P < 0.05 and **P < 0.01.

When RAW264.7 cells were stimulated by LPS, they would polarize into M1-type macrophages and release a large amount of pro-inflammatory cytokines, we detected the mRNA expression level of CD86, a macrophage M1-type polarization marker, and as shown in Fig. 12. A, the mRNA expression level of CD86 in the LPS group was significantly higher than that of the normal control group (*p* < 0.01); its expression level was significantly reduced after the intervention of adding taurine. Subsequently, as shown in Fig. 12B-D, the mRNA expression levels of IL-1β, TNF-α, and iNOS in the LPS group were significantly higher compared with the normal control group (*p* < 0.05), and the mRNA expression levels of IL-1β, TNF-α, and iNOS were significantly lower after the addition of taurine intervention (*p* < 0.05).

### Taurine-mediated modulation of the TLR4/NF-κB signaling pathway in LPS-stimulated RAW264.7 cells

**Fig. 13:**
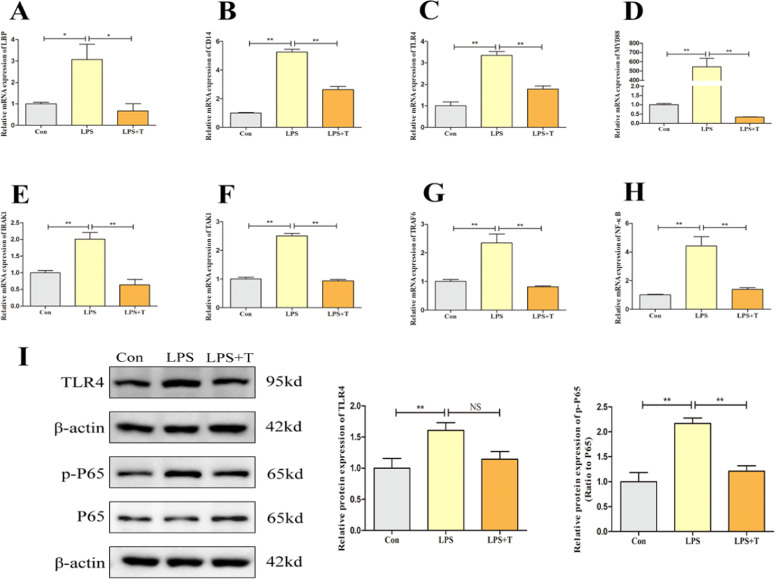
Effects of taurine on TLR4/NF-κB signaling pathway in RAW264.7 cells after LPS action. (A) Detection results of LBP mRNA expression level in RAW264.7 cells. (B) Detection results of CD14 mRNA expression level in RAW264.7 cells. (C) Detection results of TLR4 mRNA expression level in RAW264.7 cells. (D) Detection results of MYD88 mRNA expression level in RAW264.7 cells. (E) Detection results of IRAK1 mRNA expression level in RAW264.7 cells. (F) Detection results of TAK1 mRNA expression level in RAW264.7 cells. (G) Detection results of TRAF6 mRNA expression level in RAW264.7 cells. (H) Detection results of NF-κB mRNA expression level in RAW264.7 cells. (I) Western blot analysis of TLR4 and NF-κB P65 protein expression levels in RAW264.7 cells. Data were expressed as mean ± SEM. *P < 0.05, **P < 0.01, NS (P > 0.05).

In order to verify the effect of taurine on the regulation of inflammatory responses through the TLR4/NF-κB signaling pathway, we examined the changes of TLR4/NF-κB signaling pathway-related factors after LPS and taurine treatment by culturing RAW264.7 cells in vitro. Consistent with the results of the in vivo test, as shown in Fig. 13, the mRNA expression levels of LBP, CD14, TLR4, MyD88, IRAK1, TRAF6, TAK1, and NF-κB were significantly higher in the LPS group compared with the normal control group (*p* < 0.05), and the mRNA expression levels of these factors were significantly lower after the addition of taurine intervention (*p* < 0.05). Then, the protein expression of TLR4, NF-κB P65 were examined and consistent with the mRNA level, the protein level of TLR4 in the LPS group compared with the normal control group was significantly increased (*p* < 0.01); the p-P65 to P65 ratio was significantly increased (*p* < 0.01), and the protein expression level of TLR4 was decreased after the intervention of adding taurine (p>0.05), and at the same time the p-P65 to P65 ratio decreased significantly (*p* < 0.01).

### Taurine-mediated modulation of the JAK2/STAT1 signaling pathway in LPS-stimulated RAW264.7 cells

**Fig. 14:**
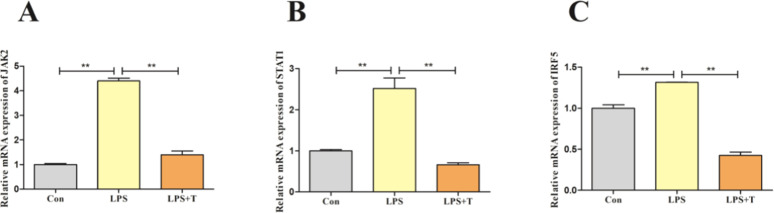
Effects of taurine on JAK2-STAT1 signaling pathway in RAW264.7 cells after LPS action. (A) Detection results of JAK2 mRNA expression level in RAW264.7 cells. (B) Detection results of STAT1 mRNA expression level in RAW264.7 cells. (C) Detection results of IRF5 mRNA expression level in RAW264.7 cells. Data were expressed as mean ± SEM. *P < 0.05, **P < 0.01.

The JAK2-STAT1 signaling pathway is an important pathway in the regulation of macrophage polarization to M1 type. Therefore, the factors related to the JAK2-STAT1 signaling pathway were examined, and the results were shown in Fig. 14. The mRNA expression levels of JAK2, STAT1, and IRF5 were significantly higher in the LPS group compared with the normal control group (*p* < 0.01), and the mRNA expression levels of JAK2, STAT1, and IRF5 were significantly lower after the addition of taurine intervention (*p* < 0.01).

### Taurine modulates NLRP3 inflammasome activation in LPS-stimulated RAW264.7 cells

**Fig. 15:**
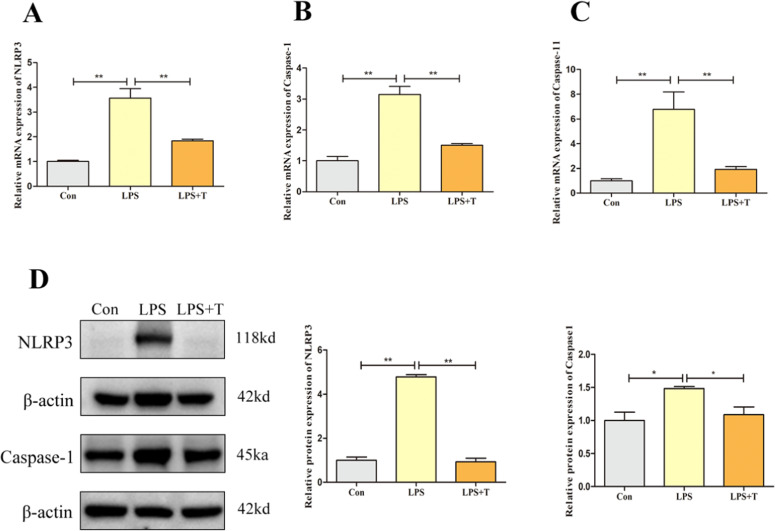
Effects of taurine on NLRP3 inflammatory in RAW264.7 cells after LPS action. (A) Detection results of NLRP3 mRNA expression level in RAW264.7 cells. (B) Detection results of Caspase-1 mRNA expression level in RAW264.7 cells. (C) Detection results of Caspase-11 mRNA expression level in RAW264.7 cells. (D) Western blot analysis of RAW264.7 cell NLRP3 and Caspase-1 protein expression levels. Data were expressed as mean ± SEM. *P < 0.05 and **P < 0.01.

By culturing RAW264.7 cells in vitro and detecting the changes of NLRP3 inflammatory vesicle-associated factors after LPS and taurine treatment, we found that in agreement with the results of the in vivo test, as shown in Fig. 15, the mRNA expression levels of NLRP3, Caspase-1, and Caspase-11 in the LPS group compared with the normal control group were significantly higher (*p* < 0.01). The mRNA expression levels of NLRP3, Caspase-1, and Caspase-11 were significantly reduced after the intervention of adding taurine (*p* < 0.01). Subsequently, the changes of NLRP3 inflammatory vesicle-associated proteins were examined, as shown in the Fig. 15D, the protein expression levels of NLRP3 and Caspase-1 in the LPS group were significantly higher compared with the normal control group (*p* < 0.05), and the protein expression levels of NLRP3 and Caspase-1 were significantly lower after the intervention of adding taurine (*p* < 0.05).

## Discussion

FLHS is a common nutritional and metabolic disease in laying hens during the peak laying period, due to high energy in the diet leading to abnormal metabolism, a large amount of fat exceeding the normal metabolic capacity of the liver is deposited in the liver, which in turn triggers liver dysfunction, leading to hepatic lipoatrophy, hepatic hemorrhage, and even necrosis. The shortening of the peak laying period, the decline in egg production, respiratory distress, and the increase in mortality rate of the laying hen will cause great economic losses and seriously limit the sustainability of the laying hen industry. Although the pathogenesis of FLHS in laying hens is still unclear, FLHS is similar to MAFLD with respect to the etiology and clinical symptoms, and some studies have shown that MAFLD can lead to pulmonary dysfunction associated with lung inflammation, and MAFLD is independently correlated with pulmonary dysfunction (Cho et al., 2022). During the pathogenesis of MAFLD, the intestinal microbiota is imbalanced, and the intestinal barrier is disrupted, leading to the translocation of LPS into the bloodstream and thus causing oxidative stress and inflammatory responses in the liver (Mokgalaboni et al., 2021; Safari and Gérard, 2019). At the same time, LPS and inflammatory cytokines also reach the lungs via the intestinal-pulmonary axis, triggering oxidative stress and inflammatory responses in the lungs, exacerbating lung damage, leading to lung dysfunction, and aggravating the condition of FLHS laying hens (Liu et al., 2021; Lv et al., 2024; Tan et al., 2020).The results of this experiment showed that the serum LPS content of FLHS laying hens was extremely significantly increased, and HE staining of paraffin sections of lung tissues revealed that the interstitial space of lung tissues was increased, and the alveolar structure was disorganized along with obvious inflammatory cell infiltration, which indicated that the lung tissues and structures of FLHS laying hens were damaged. After the addition of taurine, the serum LPS content of FLHS laying hens was significantly reduced, the alveolar structure was nearly intact, and the inflammatory cell infiltration was significantly alleviated. Taurine was found to have intrinsic antioxidant and anti-inflammatory properties, which could alleviate lung injury induced by LPS by inhibiting oxidative stress and inflammatory responses in the lungs (Bhavsar et al., 2009; Ma et al., 2022). Although the animals of the above studies were different from those of the present trial, the results of taurine alleviation of lung injury were consistent with those of the present trial, suggesting that taurine can alleviate lung injury induced by a variety of factors.

When LPS reaches the lungs, it causes oxidative stress and inflammation response in the lungs, oxidative stress can promote the expression of pro-inflammatory genes, inflammatory cytokines can promote the production of ROS, and interaction between oxidative stress and inflammatory cytokines would form a vicious circle to aggravate the progression of the disease (Miao et al., 2023; Tasaka et al., 2008). When oxidative stress occurs in the organism, enzymatic and non-enzymatic antioxidant systems interact with each other to prevent the organism from oxidative stress damage (Guo et al., 2021; Senoner and Dichtl, 2019; Zhang et al., 2019). SOD, CAT, and GSH-Px are three important endogenous free radical scavengers in the organism, which can resist oxidative damage by scavenging the excess of free radicals in the organism. MDA is a direct marker for the occurrence of oxidative stress and has a direct scavenging effect on oxygen free radicals in the organism, and the more severe the oxidative stress in the organism, the higher the level of MDA (Wang et al., 2021). In this study, the in vivo test was conducted to collect the lung tissues of FLHS laying hens, and the in vitro test was conducted to induce macrophage inflammation model by LPS, and intervened by adding taurine to investigate the effect of taurine on oxidative stress in the lungs of FLHS laying hens. The results showed that the MDA content in the lungs and RAW264.7 cells of FLHS laying hens was significantly increased, while the activities of SOD, CAT, and GSH-Px were significantly decreased, indicating that significant oxidative stress occurred in the lungs and RAW264.7 cells of FLHS laying hens; after the intervention of adding taurine, the MDA content was reduced, and the activities of SOD, CAT, and GSH-Px were significantly improved. Bhavsar TM and others found that taurine could alleviate LPS-induced oxidative stress in the lungs by decreasing lipid peroxidation and increasing the activity of lung antioxidant enzymes (Bhavsar et al., 2010), and Ommati and others found that taurine significantly alleviated oxidative stress in the lungs and mitigated histopathological changes in the lungs after taurine was added to a rat model of cholestatic lung injury (Ommati et al., 2022). Combined with the results of this experiment, it was inferred that taurine could improve the body's antioxidant capacity, reduce lung oxidative stress, and alleviate lung oxidative stress injury in FLHS laying hens.

Nrf2 signaling pathway plays an important role in maintaining oxidative stress and redox homeostasis of the organism, and it is believed that Nrf2 signaling pathway is a defense mechanism of the organism against external stimuli, can sense the state of cellular oxidative stress and regulate redox homeostasis, and its activation is conducive to the reduction of oxidative stress damage to the organism or cells (Murakami et al., 2023). Nrf2 regulates the oxidative stress response of the organism by regulating the downstream genes, such as NQO1 and HO-1. NQO1 has the unique property of clearing ROS through multiple mechanisms, and it can directly scavenge superoxide and indirectly reduce ROS by reducing antioxidants such as vitamin E, as an antioxidant role; when the body is subjected to oxidative stimuli, the expression of HO-1 rises significantly, it can be scavenged through the scavenging of ROS, peroxides, and superoxide radicals to alleviate oxidative stress (Loboda et al., 2016; Siegel et al., 2018). The results of this experiment showed that the taurine could increase the mRNA expression levels of Nrf2, NQO1 and HO-1. And the macrophage inflammation model induced by LPS showed the same results. This suggests that taurine can enhance the antioxidant capacity of lungs and macrophages of FLHS laying hens by activating the Nrf2 signaling pathway. Wu Q, Jiang Y and others found that FLHS laying hens and NAFLD rats also showed decreased antioxidant capacity during fatty liver development (Jiang et al., 2015; Wu et al., 2019); Goc Z, Zhu W and others found that alcohol exposure in the livers of mice as well as non-alcoholic fatty liver rats had different degrees of oxidative stress, and the addition of taurine increased the activity of antioxidant enzymes in the liver and decreased MDA levels, thus alleviating the oxidative stress in the liver (Goc et al., 2019; Zhu et al., 2017). Combined with the results of this experiment, it suggests that taurine improves lung antioxidant and alleviates lung injury in FLHS laying hens, and the improvement and alleviation are closely related to the activation of Nrf2 signaling pathway.

Impairment of the intestinal barrier in FLHS laying hens results in the intestinal microbial product LPS entering the bloodstream via the intestinal-pulmonary axis to reach the lungs, causing an inflammatory response in the lungs. The inflammatory response leads to the secretion of large amounts of inflammatory cytokines, generating a storm of inflammatory cytokines, leading to an imbalance in the inflammatory response and the recruitment of excessive macrophage infiltration, and in this way generating a vicious cycle that leads to more severe lung injury (Hu et al., 2021; Mason et al., 2017; Quinton et al., 2018). Macrophages usually behave in a resting state, and when altered in the microenvironment or stimulated by other stimuli, macrophages exhibit activation states with different functions and properties due to polarization. Macrophages undergo M1-type polarization when stimulated by LPS, while releasing large amounts of pro-inflammatory cytokines such as IL-1β, TNF-α, and iNOS. The results of this experiment showed that the expression level of CD80 mRNA, an M1-type macrophage marker, was significantly increased in the lungs of FLHS laying hens. Meanwhile, the expression level of CD86 mRNA, an M1 type marker, was also significantly increased after LPS stimulation of macrophages. But the addition of taurine reversed this phenomenon, and it suggested that taurine could inhibit the polarization of macrophages to M1 type. Lin S, Xie K and others found that taurine treatment reduced macrophage infiltration in the liver and inhibited the activation of M1-type macrophages in mice fed with a high-fat diet and liver fibrosis model mice (Lin et al., 2013; Xie et al., 2025). Subsequently, we quantified pro-inflammatory cytokines from M1-polarized macrophage. Consistent with enhanced M1 activation, the levels of iNOS, NO and IL-1β were significantly elevated in FLHS lungs. Taurine supplementation reversed this phenotype, as evidenced by significant reductions in iNOS and NO, and a non-significant decrease in IL-1β. In vitro experiments showed that taurine had the same effect as in vivo experiments. Previous studies have demonstrated taurine's anti-inflammatory properties in pulmonary pathology: Gurujeyalakshmi reported attenuated IL-1β and TNF-α levels in bronchoalveolar lavage fluid following bleomycin-induced injury by taurine, while Zhao observed similar suppression of these pro-inflammatory cytokines in LPS-challenged rat lung tissue (Gurujeyalakshmi et al., 2000; Zhao et al., 2022). Based upon these findings and these experimental results, we propose that taurine alleviates FLHS-associated lung injury in laying hens through inhibition of macrophage M1 polarization and subsequent attenuation of pro-inflammatory signaling cascades.

Upon arrival in the lungs, LPS binds to its binding protein LBP, forming the LPS-LBP complex, and translocate to soluble cluster of differentiation 14 (CD14), which specifically binds to Toll-like receptor 4 (TLR4) and induces TLR4 to interact with the articulatory molecule myeloid differentiation factor 88 (Myd88) via both dependent and nondependent pathways. Subsequently Myd88 further activates NF-κB, triggering an inflammatory cascade response leading to the release of pro-inflammatory cytokines IL-1β, TNF-α and IL-6 (An et al., 2022; Gao et al., 2021). The experimental results showed that taurine could down-regulate the mRNA expression levels of LBP, TLR4, Myd88 and NF-κB in the lungs of FLHS laying hens. The results of in vitro cell culture experiments showed that taurine down-regulate the mRNA expression levels of LBP, CD14, TLR4, Myd88, IRAK1, TRAF6, TAK1 and NF-κB after LPS stimulation. And the protein expression levels of TLR4 and NF-κB P65 were also down-regulated. Zhang C and others found that rat lung inflammation could be effectively attenuated by inhibiting the TLR4/NF-κB signaling pathway in an LPS-induced acute lung injury model; similarly, Zhang and others found that taurine reduced TLR4 and Myd88 protein expression in the lungs of rats in an acute lung injury model, and attenuated lung injury by inhibiting the TLR4/NF-κB signaling pathway (Zhang et al., 2021; Zheng et al., 2024). Combined with the results of this experiment, it suggests that one of the mechanisms by which taurine alleviates the inflammatory response in the lungs of FLHS laying hens is inhibiting the LPS/TLR4/NF-κB signaling pathway and down-regulating the release of pro-inflammatory cytokines, which in turn alleviates inflammatory lung injury in FLHS laying hens.

JAK2 is an important factor in the regulation of macrophage function, and the JAK2-STAT1 signaling pathway is an important pathway in the regulation of macrophage polarization. LPS or IFN-γ-mediated Janus kinase/signal transducer and activator of transcription (JAK2-STAT1) signaling induces the phosphorylation of JAK2, IRF5, and activates the expression of STAT1 to initiate the expression of M1-type macrophage polarization-related genes (Jablonski et al., 2015). When LPS or pathogenic microorganisms act on macrophages, transcription factors such as JAK2, STAT1, and IRF5 are up-regulated in the cells, initiating M1-type polarization and promoting the secretion of pro-inflammatory cytokines, which exacerbates inflammatory responses, and thus aggravates tissue and organ damage in the organism (Orecchioni et al., 2019). Li X and others found that inhibition of the JAK2-STAT1 signaling pathway could effectively inhibit M1-type macrophage polarization and which in turn alleviated the inflammatory response, thereby reducing LPS-induced acute lung injury in mice (Li et al., 2022). The results of this experiment showed that taurine reduced the mRNA expression levels of JAK2, STAT1 and IRF5 in the lungs of FLHS laying hens; and in vitro experiments also found taurine had the same effect. This suggests that taurine can inhibit the polarization of M1-type macrophages by suppressing the expression of factors related to the JAK2-STAT1 signaling pathway. Lee N and others found that inhibition of the JAK2-STAT1 signaling pathway inhibited LPS-induced polarization of M1-type macrophages and attenuated the expression levels of related pro-inflammatory cytokines (Lee et al., 2021; Ma et al., 2023; Wan et al., 2025). Therefore, we suggest that taurine inhibits the JAK2-STAT1 signaling pathway, decreases the level of polarization of M1-type macrophages, and reduces the release of pro-inflammatory cytokines thereby attenuating the inflammatory response of lungs in FLHS laying hens.

Studies have shown that LPS, when recognized by pattern recognition receptors on the cell membrane, not only stimulates NF-κB signaling but also enhances the expression and synthesis of NLRP3 inflammatory. The activation of NLRP3 mediates the downstream inflammatory response, and the overactivation of NLRP3 inflammatory is closely associated with the development of a variety of lung diseases for example the lung injury, pulmonary fibrosis, chronic obstructive pulmonary disease (Grailer et al., 2014; Hosseinian et al., 2015; Lee et al., 2016; Zhang et al., 2023). Studies have shown that NLRP3 protein is expressed in macrophages, especially expressed in large quantities in M1-type macrophages, and NLRP3 inflammatory can be activated through two pathways, the classical pathway or the atypical pathway (Awad et al., 2017). In the classical pathway, the NF-κB signaling pathway is first activated through innate immune signaling by cytokine receptors (for example TNF-α and/or TLR4-MyD88), which activates the promotion of NLRP3, pro-caspase-1, and pro-IL-1β transcripts; subsequently, the oligomerization of NLRP3 with the ASC leads to the activation of caspase-1 and the release of IL-18 and IL-1β. In the atypical pathway, Gram-negative bacteria can increase IL-18, IL-1β, IRF-3, and IRF-7 gene expression by triggering TLR4-MyD88 via NF-κB induction. IRF3-IRF7 complex induces the expression of interferon-α/β leading to activation of the JAK/STAT pathway and its downstream transcription of the Caspase-11 gene (Pellegrini et al., 2017; Rathinam and Fitzgerald, 2016). The results of this experiment showed that taurine reduced the mRNA expression levels of NLRP3, Caspase-1 and Caspase-11 in the lungs of FLHS laying hens. In vitro cell culture experiments showed that taurine not only reduced the mRNA expression levels of NLRP3, Caspase-1 and Caspase-11 after LPS stimulation, but also down-regulated the protein expression levels of NLRP3 and Caepase-1. Qiu T and others showed that taurine supplementation could reduce the protein expression of NLRP3, ASC and Caspase-1 in lung and liver tissues to reduce the inflammatory response (Liu et al., 2019; Qiu et al., 2018; Shan et al., 2024). Similarly, the results of this experiment showed that taurine inhibits the inflammatory response in the lungs of FLHS laying hens by reducing the levels of pro-inflammatory cytokines in the lungs and macrophages by inhibiting the NLRP3 inflammasome, thus alleviating the lung injury in FLHS laying hens.

## Conclusions

In summary, taurine can alleviate lung injury in FLHS laying hens, and its mechanism may be involved in improving lung antioxidant capacity by activating the Nrf2 signaling pathway, and attenuating inflammatory response mediated by M1-type macrophage polarization though inhibiting the LPS/TLR4/NF-κB, JAK2/STAT1 signaling pathway, and NLRP3 inflammasome. These results provide a theoretical basis for the application of taurine to combat lung injury in FLHS laying hens.

## CRediT authorship contribution statement

**Guangyi Ouyang:** Writing – review & editing, Writing – original draft, Data curation. **Weiwei Li:** Writing – review & editing, Writing – original draft, Validation, Data curation. **Wenke Sun:** Validation, Data curation. **Jishuang San:** Validation, Data curation. **Meichao Dai:** Validation, Data curation, Conceptualization. **Pingping Wei:** Validation, Data curation. **Jiancheng Yang:** Supervision, Resources, Formal analysis, Data curation, Conceptualization. **Gaofeng Wu:** Supervision, Formal analysis, Data curation, Conceptualization.

## Disclosures

No conflict of interest exits in the submission of this manuscript, and manuscript is approved by all authors for publication.

## Appendix Supplementary materials

Image, application 1
