# Supplementary material for: Alleviation of taurine on lung injury in fatty liver hemorrhagic syndrome laying hens by improving antioxidant and anti-inflammatory capacity
Source: Poult Sci. 2025 Dec 2;105(1):106184. doi: 10.1016/j.psj.2025.106184 (PMC12752545; doi:10.1016/j.psj.2025.106184)
Supplement: Supplementary file 1 [file mmc1.docx]

TLR4:

Line1: marker Line2: Con1 Line3: LPS1 Line4:LPS+T1 Line5: marker

Line6: marker Line7:Con2 Line8: LPS2 Line9:LPS+T2 Line10: marker

Line11: marker Line12:Con3 Line13: LPS3 Line14: LPS+T3 Line15: marker





TLR4-β-actin:

Line1: marker Line2: Con1 Line3: LPS1 Line4:LPS+T1 Line5: marker

Line6: marker Line7:Con2 Line8: LPS2 Line9:LPS+T2 Line10: marker

Line11: marker Line12:Con3 Line13: LPS3 Line14: LPS+T3 Line15: marker





p-P65:

Line1: marker Line2: Con1 Line3: LPS1 Line4:LPS+T1 Line5: marker

Line6: marker Line7:Con2 Line8: LPS2 Line9:LPS+T2 Line10: marker

Line11: marker Line12:Con3 Line13: LPS3 Line14: LPS+T3 Line15: marker





p-P65-β-actin:

Line1: marker Line2: Con1 Line3: LPS1 Line4:LPS+T1 Line5: marker

Line6: marker Line7:Con2 Line8: LPS2 Line9:LPS+T2 Line10: marker

Line11: marker Line12:Con3 Line13: LPS3 Line14: LPS+T3 Line15: marker





P65:

Line1: marker Line2: Con1 Line3: LPS1 Line4:LPS+T1 Line5: marker

Line6: marker Line7:Con2 Line8: LPS2 Line9:LPS+T2 Line10: marker

Line11: marker Line12:Con3 Line13: LPS3 Line14: LPS+T3 Line15: marker





P65-β-actin:

Line1: marker Line2: Con1 Line3: LPS1 Line4:LPS+T1 Line5: marker

Line6: marker Line7:Con2 Line8: LPS2 Line9:LPS+T2 Line10: marker

Line11: marker Line12:Con3 Line13: LPS3 Line14: LPS+T3 Line15: marker





NLRP3:

Line1: marker Line2: Con1 Line3: LPS1 Line4:LPS+T1 Line5: marker

Line6: marker Line7:Con2 Line8: LPS2 Line9:LPS+T2 Line10: marker

Line11: marker Line12:Con3 Line13: LPS3 Line14: LPS+T3 Line15: marker





NLRP3-β-actin:

Line1: marker Line2: Con1 Line3: LPS1 Line4:LPS+T1 Line5: marker

Line6: marker Line7:Con2 Line8: LPS2 Line9:LPS+T2 Line10: marker

Line11: marker Line12:Con3 Line13: LPS3 Line14: LPS+T3 Line15: marker





Caspase-1:

Line1: marker Line2: Con1 Line3: LPS1 Line4:LPS+T1 Line5: marker

Line6: marker Line7:Con2 Line8: LPS2 Line9:LPS+T2 Line10: marker

Line11: marker Line12:Con3 Line13: LPS3 Line14: LPS+T3 Line15: marker





Caspase-1-β-actin:

Line1: marker Line2: Con1 Line3: LPS1 Line4:LPS+T1 Line5: marker

Line6: marker Line7:Con2 Line8: LPS2 Line9:LPS+T2 Line10: marker

Line11: marker Line12:Con3 Line13: LPS3 Line14: LPS+T3 Line15: marker
